# Supplementary material for: Chromothripsis during telomere crisis is independent of NHEJ, and consistent with a replicative origin
Source: Genome Res. 2019 May;29(5):737–49. doi: 10.1101/gr.240705.118 (PMC6499312; doi:10.1101/gr.240705.118)
Supplement: Supplemental Material [file supp_gr.240705.118_Supplemental_file_1.zip › contigs/annotated_contigs/DB55/contig.2.DB55_length_319_mean_cov_6.11285266458.docx]

**DB55_length_319_mean_cov_6.11285266458**

```
GAGCTTGCAGTGAGCCGAGATCGCGCCACTGCACTCCAGCCTGGGCAACAGAGTGAGATTCCGTCTCAAAAAAAAAGAAGAAGAAAAAA
 >chr6:162885793-162886024 + E=4e-123
AGAAAAAGAAAGAAATTTGTGTATAAATACAAGTTTTAAGAGCCCCCCCTCCTCCTTCTTAATAAATATTAATGCATTTGCTCCTCCAC

TAAAAACACTTTAAGAAAATCAACAGCCATGGCGATCTCATGGCTGTGATG|T|CAAAGATCCCATTAATGTTTATTACAACCCCAATA
                                                    >chr6:162961420-162961501 + E=2e-34
ATATAGGTAGGCAGAATAGAATCCTGAAAAAGGTGCCAAAAGTTG|TAAAGCTGT
```
